# Exploring the lived experiences of participants and facilitators of an online mindfulness program during COVID-19: a phenomenological study

**DOI:** 10.3389/fpubh.2023.1278725

**Published:** 2023-12-12

**Authors:** Ashley Melvin, Christopher Canning, Fariha Chowdhury, Sarah Hunter, Soyeon Kim

**Affiliations:** ^1^Waypoint Centre for Mental Health Care, Waypoint Research Institute, Penetanguishene, ON, Canada; ^2^Department of Psychiatry, University of Toronto, Toronto, ON, Canada; ^3^Research and Innovation, Georgian College of Applied Arts and Technology, Barrie, ON, Canada; ^4^Department of Psychiatry and Behavioural Neurosciences, McMaster University, Hamilton, ON, Canada

**Keywords:** healthcare workers, COVID-19, mindfulness, mental health, resilience, interpretative phenomenological analysis

## Abstract

The coronavirus pandemic (COVID-19) has placed incredible demands on healthcare workers (HCWs) and adversely impacted their well-being. Throughout the pandemic, organizations have sought to implement brief and flexible mental health interventions to better support employees. Few studies have explored HCWs’ lived experiences of participating in brief, online mindfulness programming during the pandemic using qualitative methodologies. To address this gap, we conducted semi-structured interviews with HCWs and program facilitators (*n* = 13) who participated in an online, four-week, mindfulness-based intervention program. The goals of this study were to: (1) understand how participants experienced work during the pandemic; (2) understand how the rapid switch to online life impacted program delivery and how participants experienced the mindfulness program; and (3) describe the role of the mindfulness program in supporting participants’ mental health and well-being. We utilized interpretive phenomenological analysis (IPA) to elucidate participants’ and facilitators’ rich and meaningful lived experiences and identified patterns of experiences through a cross-case analysis. This resulted in four main themes: (1) changing environments; (2) snowball of emotions; (3) connection and disconnection; and (4) striving for resilience. Findings from this study highlight strategies for organizations to create and support wellness programs for HCWs in times of public health crises. These include improving social connection in virtual care settings, providing professional development and technology training for HCWs to adapt to rapid environmental changes, and recognizing the difference between emotions and emotional states in HCWs involved in mindfulness-based programs.

## 1 Introduction

The first case of Coronavirus disease (COVID-19) was reported in Wuhan, China in December of 2019 (1). By March of 2020, the World Health Organization (WHO) declared COVID-19 a global pandemic (2). Internationally, public health responses to COVID-19 included travel bans, social distancing, quarantines, school and business closures, masking policies, and the switch to virtual healthcare delivery where possible (3). Despite these measures, COVID-19 caused an unprecedented burden on healthcare systems, arising from increased workloads, shortages of personal protective equipment (PPE), distress associated with ventilator triaging, increased patient deaths, and fears about protecting oneself and their families (4–6). Taken together, these work-related stressors adversely affected the mental health and wellness of healthcare workers (HCWs) around the world (7–10). In attempts to mitigate distress, research concerning mental health interventions for HCWs became a burgeoning area of study, with evidence pointing toward online mindfulness-based programs as a promising intervention (11, 12). While there is a breadth of research concerning in-person mindfulness programs, the delivery of programming to the general public, and quantitative evaluation studies, qualitative research examining the lived experiences of HCWs participating in online mindfulness programs is lacking (13). Given that individual experiences are situated within the broader social, political, and economic dimensions of a global pandemic, further exploration into participants’ and facilitators’ lived experiences is necessary to capture nuances not previously considered (14). To our knowledge, this is the first study that utilizes a phenomenological approach to understand the lived realities of HCWs delivering or participating in an online mindfulness program during the COVID-19 pandemic.

## 2 Background

Many studies detail the toll the pandemic had and continues to have on the mental health of HCWs. Over the last few years, HCWs have reported increased symptoms of anxiety, depression, psychological distress, emotional exhaustion, burnout, moral injury, and post-traumatic stress disorder (15–22). According to a report by the Mental Health Commission of Canada, during the first few years of the pandemic, 40% of healthcare workers experienced burnout, only 60% were satisfied with the quality of care they could provide, and 50% intended to leave the profession (15). Despite the increased distress HCWs were experiencing, research in service utilization of organizational supports during the pandemic revealed that only 17.5% of HCWs opted to participate in counselling or psychotherapy services, whereas 50.4% of HCWs utilized online resources to learn about coping tools and strategies to support their mental health and well-being (23, 24). This suggests that having mental health interventions available online may help to meet HCWs’ needs and cultivate resilience (13, 25, 26).

Mindfulness is a practice rooted in Buddhism that has had a significant influence on Western science, largely through the work of Jon Kabat Zinn (27). According to Kabat Zinn, mindfulness can be defined as “the awareness that emerges through paying attention on purpose, in the present moment, and nonjudgmentally to the unfolding of experience moment by moment” (28) (p.145, par. 3). The benefits of mindfulness-based interventions are well-documented. Recent research suggests that they may help HCWs cultivate self-compassion, self-efficacy, positive emotions and increase present awareness and understanding (26, 28–30). Similar studies have found that mindfulness interventions may help to reduce stress, burnout, and depression, mitigate psychological distress, and reduce symptoms of post-traumatic stress disorder (31–33). Additionally, evidence supports the use of mindfulness-based interventions as a tool to help HCWs cope with work-related and pandemic-related stressors (34–36).

There is a growing focus on examining the effectiveness of short mindfulness-based interventions to support the mental health of HCWs during the pandemic (26, 37). While research about these interventions has yielded mixed results, a recent mixed-methods study from Kim and colleagues demonstrates that brief, online mindfulness interventions, such as the Mindfulness Ambassador Program (MAP), may improve resilience in HCWs (13). While Kim and colleagues’ study offers a brief glimpse into HCWs’ lived experiences, only participants’ experiences are considered, and facilitators experiences delivering the MAP as HCWs are largely unknown.

This paper offers a novel contribution by capturing the lived experiences of facilitators and participants in a brief online mindfulness program during the third wave of the pandemic (January/February 2021 to June/July 2021). The findings from this study can help inform program design and delivery and provide directions in tailoring mindfulness programs to the unique needs of HCWs (13). More importantly, this study elucidates the rich meanings and experiences facilitators and participants had in relation to a mindfulness-based intervention program during COVID-19.

## 3 Methodology

Using interpretive phenomenological analysis (IPA), this paper aims to elucidate the meanings that healthcare workers and facilitators attributed to their experiences in a four-week, online, synchronous delivery of an adapted version of the MAP during the first year of the pandemic. This paper deploys a relativist ontology, understanding that lived realities are expressed and understood within specific cognitive, social, and political boundaries. Our epistemological commitment is to phenomenological interpretivism, as the experiences of HCWs are culturally, socially, and historically situated, and knowledge is constituted through lived experiences. IPA was a natural methodology to select, given our commitment to exploring participants’ and facilitators’ lived experiences with MAP.

The research team made sure to situate this work within our own understanding of the connections between ontology, epistemology, and methodology and within our own positionalities in the world. Positioning epistemological perspectives is instrumental to IPA, as each researcher brings their own interpretation of individuals’ lived experiences (38). The first author (A.M.) acknowledges her position as a white, cis-gender woman, member of the LGBTQIA+ community, and undergraduate student of counselling psychology employed in research in a healthcare setting. She brings a constructivist-interpretivist epistemology with limited prior exposure to phenomenology. The co-first author (C.C.) acknowledges his position as a white, cis-gender, heterosexual man. He has a background in realist and constructivist epistemological approaches to qualitative research. While new to interpretive phenomenological analysis, his research has focused on the history of phenomenology in mental health social movements and the importance of centring lived experiences in research, practice, and policy.

The research team used the consolidated criteria for reporting qualitative research (COREQ) checklist to enhance transparency of our qualitative processes and ensure comprehensive reporting of our findings (39).

This study was approved by the Institution’s Ethics Review Board (Waypoint Center for Mental Health Care, Protocol ref. # HPRA#20.07.27).

### 3.1 Recruitment and sample

We used a purposive sampling recruitment approach because it allowed us to select participants who had rich knowledge concerning a particular phenomenon (40). Participants and facilitators were recruited via email from having registered and participated in the MAP. To be eligible for participation in this study, individuals must have attended a minimum of one mindfulness session, be aged 18 or older, and be actively employed in either direct care or indirect care roles within the healthcare sector. The sample (n = 13) was comprised of participants (n = 9) and program facilitators (n = 4), aligning with the smaller sample sizes utilized in IPA (40). All participants were women, with 69% between the ages of 31 and 50 years old, 46% employed in a non-clinical support position, and 46% employed in a direct patient care role. Only one participant worked in virtual care during the COVID-19 pandemic. Participant descriptions are outlined in Table 1.

**Table 1:** Participant information.

**#**	Age group	Gender	Current healthcare worker status	Role	Years of healthcare work experience
P1	31–50	F	Yes	Virtual Care	<11
P2	>65	F	Yes	Non-clinical support	>20 yrs
P3	31–50	F	Yes	Non-clinical support	>20
P4	31–50	F	Yes	Direct patient care	>20
P5	31–50	F	Yes	Direct patient care	11–20
P6	31–50	F	Yes	Non-clinical support	N/A
P7	51–65	F	Yes	Non-clinical support	N/A
P8	<30	F	Yes	Direct patient care	<11
P9	31–50	F	Yes	Direct patient care	<11
F1	<30	F	Yes	Direct patient care	<11
F2	31–50	F	Yes	Non-clinical support	<11
F3	31–50	F	Yes	Direct patient care	<11
F4	31–50	F	Yes	Non-clinical support	11–20

### 3.2 Data collection

After giving consent, interviewees were provided access to a secure Zoom link, and interviews took place from October 2020 to March 2021. Interviews were scheduled for 60 min to allow enough time to gain a rich understanding of their experiences in the program. Interviews were guided by a semi-structured interview protocol. The protocol was developed in collaboration with the advisory committee, which was composed of the research team and experienced mindfulness practitioners. The advisory committee started with the following two overarching questions:

1. What was the role of the online mindfulness program on social connectedness, empathy, and resiliency during the COVID-19 pandemic?
2. What were the experiences of both participants and certified facilitators delivering the online course to strengthen the online facilitation?

Drawing upon these overarching questions, the advisory committee developed five open-ended interview questions to guide meaningful conversations about participants’ and facilitators’ lived experiences. Semi-structured interview questions were designed with rapport-building at the forefront of each interview (40). Questions were limited to allow participants to articulate their experiences in detail. Sample participant questions included:

1. Can you tell me a bit about the impact of the pandemic on your overall physical and mental well-being?
2. What drew you to the four-week mindfulness program? What were you hoping to learn or experience in the program?
3. How do you feel the four-week program impacted your overall well-being and your work performance?

Sample facilitator questions were:

1. What is your experience facilitating mindfulness programs, both online and face-to-face?
2. What challenges, if any, did you experience as a facilitator when you moved to deliver the program in-person to online?
3. How would you describe the well-being of the participants at the onset of your course?

All interviews were conducted by S.H. and recorded with the consent of participants for the purpose of transcription. At the end of the interview, participants were sent an honorarium in the form of a $25 gift card. Interviews were transcribed verbatim, and participant numbers were assigned to uphold confidentiality. All transcriptions were conducted using Nvivo and were checked by the research team (F.C. and S.H) for accuracy, quality, and integrity and were sent back to each participant for member-checking. At this time, participants had the opportunity to layer in detail and expand on experiences.

### 3.3 Data analysis

Participant interviews were analyzed using IPA (41). To adhere to the idiographic and reflexive nature of IPA, each author acknowledged their own epistemological views and practiced reflexivity throughout all phases (41). During phase one, A.M. and C.C. immersed themselves in the data by reading and re-reading the transcripts multiple times. In phase two, A.M. and C.C. took descriptive, linguistic, and conceptual notes of each transcript separately. In phase three, A.M. and C.C. independently identified emergent themes within each case. In phase four, A.M. and C.C. reviewed, compared, and connected themes. Each author made connections between themes within each case and started to review, synthesize, and connect themes. Phases one through four were repeated for each participants’ transcript, with bracketing used between participant transcripts. A summary of these emergent themes can be found in Table 2. In phase five, the team used cross-case analysis to identify and prioritize patterns, including convergent and divergent themes, a practice that can be used when analyzing multiple interviews in IPA (42). A.M. and C.C. independently conducted a cross-case analysis, noticing commonalities of themes across all participants. In phase six, A.M. and C.C. shared results from the cross-case analysis, finalized themes inductively, and identified quotes that provided rich descriptions of the shared patterns of experiences, forming the interpretation of the final themes. A summary of this process can be found in Table 3.

**Table 2:** IPA emergent themes.

Participant	IPA Emergent themes (C.C.)	IPA Emergent themes (A.M.)
P1	Zoom fatigue The connection between burnout and isolation Overcoming isolation through connection Importance of routine Overwhelm, fatigue, stress	Overwhelm and Zoom fatigue Scheduled self-care Community and connection refreshing Offset challenges at work Emotional shift
P2	Isolation and loneliness Compounding life stressors Mindfulness helped when they felt they were “losing control” Sense of connection “to a point” in online spaces	Loneliness, fear, guilt Connection but limited Facilitated use of self-help Grounding experience
P3	Connection over Zoom Support from others Many challenging emotions piled up	Feelings compounded over time Work and mood changed Connection to others through activities Emotional/mindset shift More control
P4	Loss of community and connection Looking for coping strategies Challenges with COVID	Change in work environment and in activity levels Self-care is needed and important Connection was limited
P5	Stressed that mindfulness was so hard Never knew anxiety before The course did not do what they had hoped or wanted Feeling overwhelmed Doing what they could to get by	Stressed about mindfulness, work, and challenges at home Heightened guilt, anxiety, and stress Online program fell flat
P6	Shared community Connection through mindfulness practice Just doing what you could Virtual saved time	Constant change Mindfulness to cope Connection needs met Structure, routine, schedule Enjoyed the structure of the sessions Connected to the practice
P7	Stress with work and family during COVID Program short but took small things from it Didn’t find social connection with participants Very social person	Separating work from home challenging Community/connection lacking Program too brief Structure was beneficial
P8	Complicated grief Complex and compounding emotions Lack of attention Course reminded them to pay attention and do what they can to focus on their mental health	Increasing anxiety, fatigue, exhaustion Lots of goodbyes Chaos, falling apart, relentless No connection, felt alone It was hard to practice mindfulness Timing was difficult Interruptions/distractions Operational stressors adding to stress
P9	Emotional exhaustion Changes to routine Practicing self-care Finding the time to maintain the practice Lacking a sense of community yet longed for connection Resilience is in connection	Changing work environment No routine Struggling to function Self-care, routine, back on track. No community Community is self-care
F1	Importance of self-care Bearing witness to suffering and burnout Finding connection in whatever way possible Zoom challenges Do what you can for self care	Uncertain about program reach Observed burnout and emotional distress Planting seeds of self-care
F2	Disconnect over Zoom/Zoom challenges Witness to so much burnout but that people were doing what they could Witness to resilience Shared experiences Different forms of connection	People left better than they came People are resilient Facilitators were also resilient Connection present, but limited Zoom challenges
F3	Difficulties with online and family life Compounding emotional toll of participants Zoom challenges Importance of care of the self Found connection with participants	Advantages and disadvantages Connection different online Recognized role of organization Technology issues Timing of program difficult
F4	Caring for the self and doing what you can Witness to a lot of stress and big emotions Togetherness emerged in the sharing of emotions in a shared space Zoom challenges Some disconnect with lack of real time feedback Loss of “essence” of the program	Participants were in a sensitive place Resilience/self-lessness Community through Zoom chat Zoom issues Facilitation was not as natural Benefited personally and professionally

**Table 3:** IPA analytic process.

Emergent themes	Cross-case analysis: superordinate and subordinate themes				Final themes
	Work, home, and online life	Complex emotions and states	Community and connection	Doing what you can	
Case-by-case IPA (C.C.; see Table 2)	Shared environments Uncertainty and isolation Everything is changing, falling apart Strains on healthcare systems Hectic work and family lives Technology/Zoom challenges Challenges and benefits of the online switch	Heightened and overlapping emotions Complex emotional states Concern for self and others Witness to suffering Compounding emotions	Connection between facilitators and participants Connection between participants Facilitators fostering connection Something was missing Lack of community, connection, & accountability in online life	Integrating mindfulness into daily life Maintaining a sense of normalcy Simple practices and tools Structure & routine Acceptance Small steps towards self-care	Changing environments
					Snowball of emotions
Case-by-case IPA (A.M.; see Table 2)					Connection and disconnection
					Striving for resilience

## 4 Findings

The methodological approach utilizing IPA resulted in four themes: (1) changing environments; (2) snowball of emotions; (3) connection and disconnection; and (4) striving for resilience, defined as “a dynamic process encompassing positive adaptation within the context of significant adversity.” (43) (p.543, par. 1).

### 4.1 Theme 1: changing environments

The first theme that emerged was *changing environments*. This was derived from the lived experiences of participants and facilitators as their external environments shifted dramatically in response to the COVID-19 pandemic. The pandemic increased work-related stressors leading up to those taking the mindfulness-based program and seeking support. For example, Participant 7 said, “It is stressful, and it is challenging, especially when you have got COVID-positive patients, and you want to make sure that you are doing everything properly to protect yourself.” Similarly, as the pandemic progressed, caring for patients changed and bearing witness to suffering was very challenging. As Participant 8 said:

You would hold the video for someone who is dying of COVID, and at the time, family was just not allowed to come in if you were dying. But now, in wave three, I’m holding the video for someone dying of it, if I even can, because not only does the whole family have COVID, but half of their family is in the hospital.

Participant 8 went on to describe the stress that arose out of inconsistent institutional policies and practices and the effect these had on patients’ families who were caught in the middle:

Being in a role where I'm on all the units in the hospital, I really struggle with different interpretations from different managers the frustration of families whose loved one gets moved between different floors. They were allowed to visit twice a week and then they're not allowed to visit at all just because different managers [manage] differently.

Two facilitators also experienced changes in their work environment as the mindfulness-based program transitioned to online delivery. This transition was a steep learning curve for some. Facilitator 4 stated: “The biggest challenge was sorting out how to use the camera on Zoom while referring to your notes.” Facilitator 2 echoed this when discussing their experiences in learning how to use software to deliver the program: “Using PowerPoint to … guide people through mindfulness is hard … it’s just not natural.” Facilitator 2 also explained that participants’ technological issues were disruptive to the mindfulness sessions: “Participants [had] technological challenges and [reached] out to me thinking I can kind of solve that for them [and] added obstacle[s] to having successful sessions.”

Although the online switch was challenging, facilitators also shared how they adapted and supported each other during this transition. Facilitator 4 detailed: “One of the things we did that I think was helpful for the facilitators was just booking some mock sessions in advance. We also incrementally increased what they needed to do while they were delivering.” Facilitator 4 explained further that part of adapting was adjusting their expectations for participants:

Part of the communication I made was letting people know they had permission to have no camera on, and that was important. Some people even would put a different name up and then would privately let me know who was there like they wanted to be anonymous.

### 4.2 Theme 2: snowball of emotions

The second theme that emerged from our analysis was the *snowball of emotions*. This theme describes the complicated, overlapping, and accumulating emotions and emotional states participants and facilitators experienced leading up to and participating in the program. Participants and facilitators often spoke about specific emotions (e.g., fear, sadness, worry) interchangeably with emotional states (i.e., anxiety, fatigue, and burnout). In our discussion below, we differentiate between the two to provide a richer interpretation of these complex experiences.

Participant 5 experienced anxiety, which was new and distressing for them: “I’ve never really had anxiety before. Now … my thoughts are a lot more disorganized.” Participant 1 described compounding effects of exhaustion, uncertainty, and overwhelm: “I think there’s … emotional exhaustion, like worrying about people … [and] more uncertainty in terms of working with people in crisis.” These complicated emotions led to burnout for some participants and were a reason they looked to the mindfulness program for support. Participant 1 explained, “I was … looking for some resources and support. If it wasn’t COVID … I might not have felt burnt out.”

The emotional toll and burnout among people in the healthcare field was evident, as Participant 8 described: “It was harder than I was expecting it to be. Probably harder emotionally and just a lot of fatigue and exhaustion and burnout and feeling that not only in myself but in my colleagues and in … all the healthcare providers I’m interacting with.” It was clear that for these participants, caring for patients amidst the pandemic when the demand was significant brought compounding challenges.

Facilitators were in a unique position experiencing their own heightened emotions while witnessing the struggle of HCWs. Facilitator 4 said, “There were times where I was really stressed and teaching virtually and the dog might bark, and things might happen, and there are many things in life going on, and there’s a lot of anxiety because you want it to be perfect.” When reflecting on how participants appeared to be struggling, Facilitator 1 also noted that there was a stark awareness of the degree of burnout among HCWs:

I think it really showed me how much impact this pandemic is making on people. Within a health-helping profession, there's always going to be stress, and there's going to be burnout. With this pandemic, I truly understood the definition of burnout and not just using it as a loose term … I was surprised how many people were staying lower on the emotions thermometer.

Facilitator 4 shared similar sentiments: “People did not want to be necessarily seen … they wanted to be heard. I noticed that piece just giving permission not to have your video on. People were present, but they were fragile. The [course] was a place for them to be safe and fragile and take what they need.”

The online MAP met participants at a time when emotions were high and when support was needed most. Facilitators saw participants’ emotional burden in sessions and aimed to create a non-judgmental environment where such vulnerabilities could be held. The facilitators’ efforts to provide care in an online environment were felt by the participants in different ways. The third theme, connection and disconnection, further explores this sense of community for some people along with barriers to togetherness for others.

### 4.3 Theme 3: connection and disconnection

The third theme that emerged from the interviews is *connection and disconnection*. Despite the many challenges within changing environments and the compounding factors that led to complex emotions and emotional states, participants and facilitators mostly saw the course as a tool for connection. Unfortunately, the course seemed to exacerbate a sense of disconnection for others who found online life challenging.

Participant 1 felt that connection came in the form of a community of HCWs seeking help amidst the pandemic out of a shared necessity: “There’s this sense of community that we all [have] coming with different struggles,” they remembered. Similarly, Participant 3 felt this sense of community, particularly with their peers within the group: “[I was] feeling connected. We were sending good thoughts to people … over Zoom.” Participant 1 had similar reflections: “The facilitator really did a good job of creating a sense of safety and community,” they recalled.

When discussing the connections they observed in the mindfulness program, Facilitator 4 shared: “I think there is a togetherness because people chose to be there. This was a choice, and using the chat box [enforced] that togetherness … I think that really made a community … the chat would fire up with support, positivity and validation for people where they were.” While Facilitator 4 noted the connection between the group members, Facilitator 3 felt that the connection was stronger between themselves and the participants directly:

I think more connection was between myself and the participants. There were, I would say, like a dozen occasions where one participant would share something and then somebody would immediately type afterwards like me too … I think probably the greater connections are the ones between myself and the repeat participants.

Although some participants and facilitators thought the program fostered community and connection, some felt disconnect in the way the program was delivered. For Participant 4, the lack of participant consistency contributed to the disconnect: “There wasn’t the consistency week to week of participants …I do not know what happened to all those people from week one.” For Participant 5, the online environment limited accountability in adopting the skills that the mindfulness program offered: “Things that require a lot of willpower and effort and … anything that requires willpower, it’s always helpful to be accountable in some way.” For Participant 8, being in a Zoom meeting with others who had their cameras off fuelled a sense of disconnection: “I did not feel a sense of connection or community in the group,” they said, “partly because they are just names on a screen that I do not actually see them or get to know who anybody is.”

Facilitator 2 described some of the factors they believed contributed to participant disconnection: “They [participants] stopped [coming] … They just did not know if it was right for them. There was nothing really holding them from showing up to this free Zoom commitment that they have made for half an hour once a week.”

While not all participants experienced connection and community in the program, the following theme demonstrates that most participants and facilitators took strides towards bettering their mental health in whatever small way they could.

### 4.4 Theme 4: striving for resilience

The final theme that emerged from participant and facilitator interviews was *striving for resilience*. Even with pandemic-related stressors, workplace demands, and challenges with the shift to online life, participants put effort into bettering their mental health. For example, Participant 1 said, “[The program] really helped to kind of put everything back into perspective, kind of close the lid on some of the feelings that would have led to burnout and taking stress leave. It did help for sure.” Others shared that the mindfulness program enhanced self-awareness, self-compassion, acceptance, and the ability to shift emotions. Participant 1 said: “Where instead of feeling overly emotional or overwhelmed, [the course] brought me back to a sense of calm.”

Some participants commented on how the program provided structure and routine, a space for them to integrate mindfulness practice into their daily lives as a practice of self-care. Participant 1 stated: “[The program] really helped me with setting a routine again and getting back into my mindfulness practice more regularly because … there was sort of this reminder.” Participant 9 shared similar thoughts:

Sometimes when you start working, the daily routines get in the way and you're not allotting time to engage in self-care … [MAP] was a reminder [and] was one of the reasons I joined … I need structure… I'm very committed as long as it's in my calendar.

According to Participant 1, self-care does not have to be complicated to be effective. “The emotion thermometer stood out to me,” they said, “because it was such a simple practice, but such a good way of checking [in]. It made such a nice thing to do it in a group format where people could kind of put in a number on a scale to give it in chat or look at colour and kind of see where they are at.”

Two facilitators also shared their experiences as bearing witness to participants aspiring to resilience. Facilitator 4 shared:

One group I worked with were people who worked with geriatrics. They knew what was coming their way. They were preparing. That was eye-opening. They were trying to really manage that emotional exhaustion they were feeling now. It showed me how incredibly strong and … how selfless they are … because they were doing it to care for themselves [and] to care for other people.

Facilitator 1 reiterated this sentiment: “[It speaks] to the resilience that people have when they are attending or seeking out these services, that they are recognizing perhaps that there’s something within them that they want to improve or maintain.” In sharing observations about participant progress in the program, Facilitator 1 continued, “I’ve noticed quite a bit of improvement [in] people’s moods, even though I’m not able to see them and see the nonverbal side of it.”

Experiences leading up to the mindfulness program, through its completion, were unique to everyone. At the same time, participants and facilitators shared a common experience: rapid environmental changes to a major world event led to heightened and complex emotions and emotional states that fuelled a desire to find relief through mindfulness.

## 5 Discussion

This study explored the lived experiences of HCWs participating in or delivering an online mindfulness program during COVID-19 and the impact of mindfulness on their personal and professional lives. The study found four themes: changing environments, snowball of emotions, connection and disconnection, and striving for resilience.

The first theme, *changing environments,* articulates how participants and facilitators experienced rapid changes to work, family, and social relationships, and how they faced challenges with the shift to online life during the first year of the pandemic. This shift posed significant challenges for both groups. Participants and facilitators, all of whom were HCWs, found themselves contending with feelings of loss, longing, and loneliness, as well as fear and grief. These findings align with existing studies detailing how fear and distress were attributed to changing workplace environments during COVID-19 (6, 44–46). For most facilitators, the switch to delivering the mindfulness program over Zoom brought frustration. This was caused by inexperience with delivering courses online, and factors such as participants having poor internet connections or trouble accessing course materials. Despite these challenges, facilitators demonstrated adaptability and supported each other. Simultaneously, many grappled with feelings of loneliness, sadness, and disconnection in other facets of their lives. Previous qualitative research echoes these findings, highlighting the difficulty of adapting to rapid changes within healthcare environments during COVID-19, whether in person or virtually. Emotional stressors were compounded by critical shortages of PPE (44, 45). Accelerated organizational changes placed an undue burden on HCWs, leaving them unprepared for increased patient demands and shifting organizational policies that influenced personal safety and the delivery of patient care (45).

The second theme, *snowball of emotions,* describes the complicated and compounding emotions participants and facilitators experienced in response to these rapidly changing environments. Every participant and facilitator spoke about how the pandemic, resulting public health measures, and changing work and social environments contributed to emotions like anger, fear, and sadness and more complex emotional states like exhaustion, isolation, and burnout, consistent with prior research (6, 8, 9, 15, 17, 22, 25). Participants described experiencing overwhelm and grief while witnessing suffering during wave after wave of the pandemic when not much was known about the virus. Participants all described anxiety about their mental health while trying to care for others in healthcare roles. They spoke openly about loneliness, isolation, disruption to routines, the compounding effect of burnout, and their desire to find mindfulness or other mental health resources to cope in response. On the other hand, most facilitators also articulated positive emotions, including empathy towards their colleagues, pride from being able to help others through mindfulness, and gratitude for fostering a sense of connection in online spaces. Similar results concerning co-occurring or gradually emerging positive emotions during public health crises have been reported in other studies (46–48). However, our finding that facilitators experienced positive emotions while delivering the online mindfulness program is unique.

The third theme, *connection and disconnection,* explores how some participants and facilitators found the program to be a space for social connection during a crisis, while others found it to be frustrating and difficult. Those who appreciated the course tended to express hope and gratitude about having a community of people with whom they could connect. Participants who found the course frustrating often expressed grief about losing in-person connections; an online course did not seem to alleviate feelings of loneliness. Nevertheless, most participants spoke positively about how the program helped offset work and family life challenges. Previous research has similarly demonstrated that social support can facilitate psychological adjustment during a pandemic (46, 49, 50). Some participants found a connection through specific program elements, such as question and answer periods, and shared reflections on gratitude. The emotions thermometer was cited most often as helping with connection because it allowed participants to express the range of emotions they were experiencing without judgment, in community. Others felt disconnected when participants changed week to week, when some people turned cameras off, or when technological issues occurred. In contrast to previous research, some participants felt that the online delivery took away from a sense of connection, exacerbating emotional states such as loneliness and burnout (51). Overall, facilitators appreciated most aspects of the program and the community it created. However, one missed the somatic and nonverbal elements of an in-person program, such as eye contact, facial expressions, and physical closeness.

The final theme, *striving for resilience*, highlights how participants and facilitators used strategies from the mindfulness program to cope. For participants, resilience materialized through routine and structure during a time that felt uncertain and chaotic. Some found ways to integrate small things they learned into their daily lives, such as breathing, gratitude, meditation, and the emotions thermometer. Almost everyone spoke about how the mindfulness program helped them learn self-care and reminded them of the importance of community. This finding aligns with research demonstrating that self-care mediates the relationship between social connection and psychological adjustment (49). However, self-care, we heard, can only take people so far before they need to connect with others and feel supported by broader social systems. This finding is consistent with research about how individual-level programs need to be coupled with institutional and structural support to prevent burnout in the workplace (52). Facilitators also felt a sense of improved resilience through self-reflection, gratitude, adaptability during technological challenges, and their commitment to mindfulness. Facilitators’ struggles were alleviated by witnessing participants being strong and selfless in doing what they could to get by, however incrementally. Overall, the online program supported participants in coping with the pandemic and provided a space for facilitators to build resilience by supporting others.

### 5.1 Study strengths and limitations

To our knowledge, this is the first study to utilize IPA to understand participants’ and facilitators’ lived experiences of an online, synchronous, four-week mindfulness program. Utilizing this methodology enabled a rich interpretation of participants’ subjective experiences of participating in the MAP when COVID-19 caused significant institutional changes, human resource challenges, and increased strains on healthcare systems worldwide. This study offers a contribution to the limited qualitative research concerning HCWs’ and facilitators’ experiences as part of a mindfulness-based program.

This study had limitations that warrant further consideration. The respondents who agreed to participate all identified as women. The homogenous nature of our sample limits our understanding of the unique experiences of HCWs of other genders. Additionally, as in all qualitative research, and especially in phenomenological research, our results are not generalizable to other healthcare workers. A third limitation of our study is that some participants struggled to recall some of the mindfulness tools discussed in the program, which may have influenced a recall bias in some participants. A final limitation of our study were the challenges we faced in conducting web-based interviews. In some cases, this format limited our ability to connect with participants and was a barrier to interpreting non-verbal cues and other contextual details of participants’ experiences.

## 6 Conclusion

The current study explored the lived experiences of HCWs who participated in and facilitated a brief, online mindfulness-based program during COVID-19. Although the results were mixed, this study demonstrates that when organizations provide HCWs with mental health resources, employees will try to improve their mental health and well-being. However, online mindfulness programs may not be effective for everyone. Additionally, wellness interventions targeted towards individual behavioural changes will only influence employee well-being to some degree. Employees require support from organizations to foster a psychologically safe and healthy workplace. To support and sustain HCW well-being, improvements must be directed at addressing discrepancies between institutional and worker concerns. A holistic approach to HCW mental health is crucial, integrating individual interventions like mindfulness with systemic support emphasizing proactive mental health initiatives in workplaces. To understand their long-term effects, continuous feedback mechanisms in programs and interventions are vital to ensure lasting benefits and adaptability in the demanding healthcare field.

As our findings produced mixed results, much is left to be learned about the benefits of online, brief mindfulness interventions for HCWs and program delivery during times of crisis. Enhancing connections in future online mindfulness-based programs will be essential. For participants who felt a sense of disconnection, consistency in the weekly participants and assigned facilitator was noted as a strategy that may be helpful in future cohorts. To ensure greater consistency, future programs could consider a closed group design. Additionally, being off-camera was cited as a barrier to developing peer-to-peer connections, whereas the option to remain anonymous drew others into the program. A possible solution would be to designate mindfulness programs specific to those who wish to participate with cameras on versus off so individuals can choose how they wish to participate. Hybrid program models that combine online and in-person sessions could also cater to diverse needs. As technology problems were a frequently cited concern with the online program, providing a space to check computer systems prior to a virtual session could help troubleshoot such issues.

Future research should explore methods for enhancing connection in virtual care settings. Future interventions can also prioritize professional development and technology training for HCWs to address rapid environmental changes promptly when they arise, along with potential barriers to adaptation. Virtual or hybrid mindfulness-based programs should emphasize a deeper understanding of specific emotions versus broader emotional states.

## Data availability statement

The raw data supporting the conclusions of this article will be made available by the authors, without undue reservation.

## Ethics statement

This study involving human participants was approved by the Institution’s Ethics Review Board (Waypoint Center for Mental Health Care, Protocol ref. # HPRA#20.07.27). This study was conducted in accordance with the local legislation and institutional requirements. The participants provided their written informed consent to participate in this study. Written informed consent was obtained from the individual(s) for the publication of any potentially identifiable images or data included in this article.

## Author contributions

AM: Formal analysis, Investigation, Methodology, Writing – original draft, Writing – review & editing. CC: Formal analysis, Investigation, Methodology, Supervision, Writing – original draft, Writing – review & editing. FC: Formal analysis, Investigation, Methodology, Writing – original draft, Writing – review & editing. SH: Investigation, Supervision, Writing – review & editing. SK: Conceptualization, Funding acquisition, Methodology, Project administration, Supervision, Writing – review & editing.
